# A case involving laparoscopic decortication of a large simple renal cyst using conventional monopolar device

**DOI:** 10.1016/j.ijscr.2022.106866

**Published:** 2022-02-25

**Authors:** Kota Kobayashi, Shuko Yoneyama, Eren Iwasa, Jurii Karibe, Daisuke Yamashita, Akitoshi Takizawa

**Affiliations:** Department of Urology, International Goodwill Hospital, 1-28-1 Nishigaoka, Izumi-ku, Yokohama, Kanagawa 245-0006, Japan

**Keywords:** CT, computed tomography, MINO, minocycline hydrochloride, Simple renal cyst, Laparoscopic decortication, Laparoscopy, Monopolar device, Symptomatic

## Abstract

**Introduction and importance:**

Simple renal cysts are common in adults, but most of them are asymptomatic. Usually, percutaneous puncture is an initial treatment, but laparoscopic decortication may be effective for recurrent simple renal cyst. Herein, we report a case in which a large symptomatic simple renal cyst was treated with laparoscopic decortication using conventional monopolar device.

**Case presentation:**

A 34-year-old female visited our hospital with chief complaints of back pain and abdominal fullness. Computed tomography showed a right simple renal cyst (diameter: 140 mm). We performed percutaneous drainage with sclerotherapy, but the cyst recurred a month later. Thus, we carried out laparoscopic decortication. We opened the cyst wall via a retroperitoneal approach and trimmed it using monopolar scissors. The operation time was 124 min. The patient's postoperative course was uneventful, and no complications were observed. Following surgery, the patient was asymptomatic.

**Clinical discussion:**

In our case, we performed operation using a conventional monopolar device without sealing devices. It has been reported that the use of sealing devices can make laparoscopic surgery safer and reduce the operation time, but we herein report that laparoscopic decortication with a conventional monopolar device is an effective and safe treatment option for symptomatic simple renal cysts and that more expensive energy sources are not required.

**Conclusion:**

We successfully performed laparoscopic decortication of a large symptomatic simple renal cyst. This operation is minimally invasive and safe.

## Highlights

• Simple renal cyst are common and most cases are asymptomatic, but large simple renal cyst may cause symptoms such as pain.
• As an initial treatment, percutaneous puncture is widely performed, but recurrence rates are high.
• Laparoscopic decortication may be an effective and safe treatment option for large symptomatic simple renal cysts.

## 1 Introduction

Simple renal cysts are common; i.e., their incidence among the general population is about 12%, and they increase in size and number with age [1]. Most cases are asymptomatic, and need no treatment, but symptoms, such as pain, hematuria, and infection, may occur due to the growth of the cyst. Usually, as an initial treatment, percutaneous puncture with or without sclerosing agent, but recurrence rates are high. Laparoscopic decortication is not very common but useful for renal cysts, as it has a higher success rate than other methods and involves minimal blood loss and a shorter operation time [2].

This case report is reported in line with the SCARE 2020 criteria. [3]

## 2 Case presentation

**Fig. 1:**
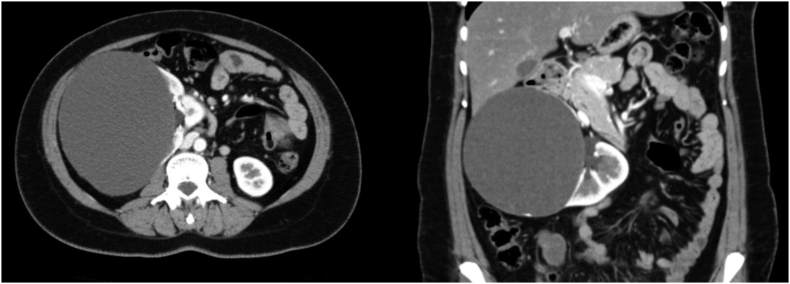
Contrast-enhanced CT showed a right simple renal cyst measuring 140 mm in diameter.

**Fig. 2:**
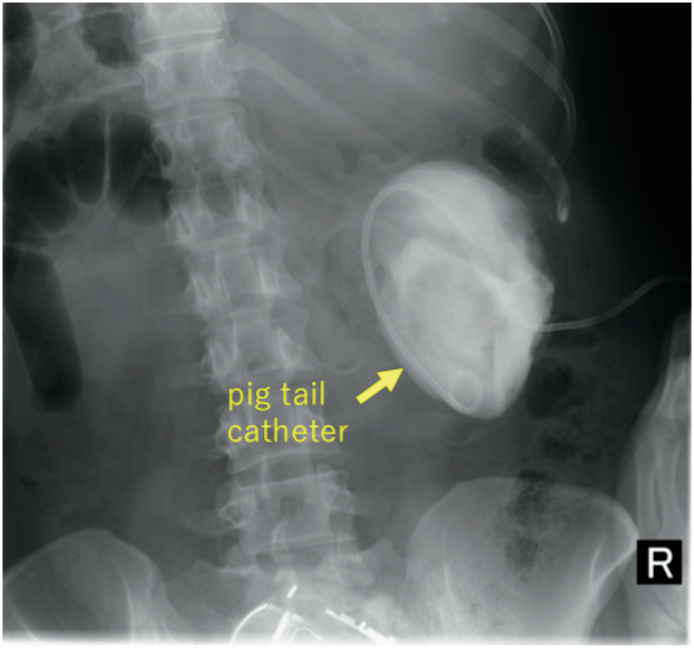
Percutaneous puncture was performed, and there was no connection between the cyst and urinary tract.

**Fig. 3:**
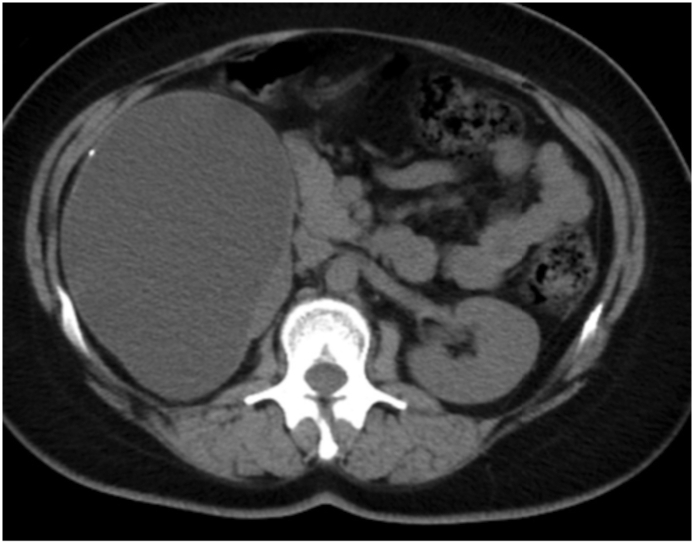
CT showed the recurrence of the renal cyst, measuring 148 mm in diameter.

A 34-year-old female, with no relevant medical or family history, visited our hospital with chief complaints of back pain and abdominal fullness. Her pain had been gradually worsening for the past 6 months and was more intense with physical movement. The intensity of the pain was about 40 mm on the Visual Analogue Scale (VAS). At her first visit, no abnormal vital signs nor physical examination were noted. Computed tomography (CT) showed a right simple renal cyst, measuring 140 mm in diameter (Fig. 1). First, we performed percutaneous drainage and sclerotherapy. We punctured the cyst under ultrasonography and inserted a pigtail catheter, and 920 ml of serous fluid was drained from the cyst. After the transcatheter injection of contrast medium, all of the contrast medium remained within the cyst, which indicated that there was no connection between the cyst and urinary tract (Fig. 2). We selected minocycline-hydrochloride (MINO) as the sclerosing agent, injected 100 mg MINO and saline using the transcatheter approach, and clamped the pigtail catheter for 10 min. Immediately after this treatment, the patient's back pain disappeared, but two months later CT showed the recurrence of the renal cyst, measuring 148 mm in diameter (Fig. 3). After another month, her back pain also reappeared. We decided that conservative management would be difficult; thus, laparoscopic decortication of the renal cyst was carried out.

**Fig. 4:**
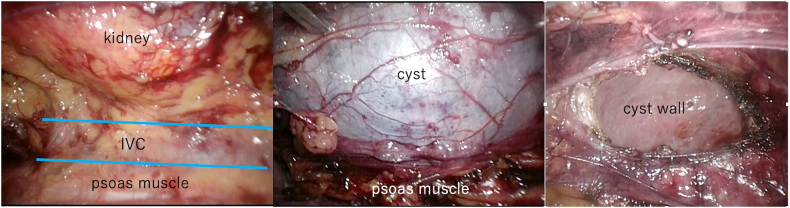
Laparoscopic decortication of the renal cyst was performed, the cyst wall was circumferentially resected using a monopolar device, and the cyst fluid was aspirated.

The operation was performed under general anesthesia. The patient was placed in the left lateral decubitus position. We created three ports, incised the lateroconal fascia via a retroperitoneal approach, and identified the cyst on the upper pole of the kidney. After visual inspection, the cyst wall was circumferentially resected using a monopolar device, and about 1200 ml of the cyst fluid was aspirated (Fig. 4).

**Fig. 5:**
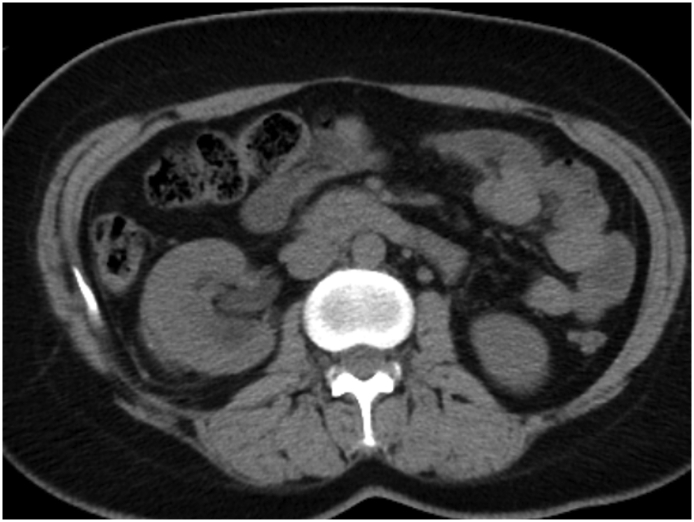
CT showed no recurrence of the renal cyst.

The operation time was 124 min. Blood loss was minimal. The patient's postoperative course was uneventful, and no complications were observed. The patient was discharged 5 days after the operation. After the operation, her pain disappeared, and at 6 months after the operation, CT showed no recurrence of the renal cyst (Fig. 5).

## 3 Discussion

Simple renal cysts are common and occur in a wide range of age groups, although their size and number increase with age [4], [5]. Most of them are asymptomatic and usually do not require treatment, but larger cysts may occasionally cause pain, infections, and/or hematuria, or rupture. There are several treatment options for symptomatic renal cysts. As an initial treatment, percutaneous puncture with or without sclerosant therapy is widely performed, and good outcomes have been reported [6], [7]. On the other hand, recurrence rates are high, and multiple punctures are often required [8]. In fact, in our case, percutaneous drainage and sclerosis were performed, but recurrence was observed soon after the procedure.

Laparoscopic decortication is a useful option for simple renal cysts. Several studies have reported that laparoscopic treatment is the first-line minimally invasive treatment for simple renal cysts and has a low recurrence rate [9], [10], [11]. Comparing both treatments, it had been reported that the success rate of percutaneous sclerotherapy was about 55%, while the success rate of laparoscopic decortication was more than 95%. [12] The Bosniak classification is used to evaluate renal cystic masses. Laparoscopic decortication is basically indicated for Bosniak classification type I renal cysts.

In patients with suspected cystic renal carcinoma, nephrectomy or partial nephrectomy should be performed, but there have been cases of renal cysts in which renal cell carcinoma was diagnosed by laparoscopic decortication, and nephrectomy was performed later [13]. Bosniak classification type II and higher cysts should be carefully evaluated before such surgery. The absence of a connection between the cyst and the urinary tract is the one of the diagnostic criteria used for simple renal cysts, but there has been a report of a renal cyst with a connection to the urinary tract, which was identified during surgery. In the latter case, the cyst wall was opened, and the fistula to the urinary tract was closed with sutures [14]. If possible, preoperative retrograde urethrography or percutaneous puncture should be carried out to confirm that there is no connection between the cyst and urinary tract, as this would make the subsequent operation safer.

In our case, we selected a retroperitoneal approach. The retroperitoneal approach involves a narrow field of view, which may limit forceps manipulation, but in our case although the renal cyst was large, there was no need for an assistant port, and left and right ports for the surgeon were sufficient for manipulation. Furthermore, it had been reported that retroperitoneal approach reduces the operation time and postoperative pain compared with the transperitoneal approach. [15], [16] The transperitoneal approach involves a wider field of view, but carries a risk of intestinal injury or postoperative ileus. If a cyst is located on the ventral side of the kidney, the retroperitoneal approach is not suitable because it is necessary to achieve sufficient renal release, and the transperitoneal approach is considered to be more suitable.

In our case, we used a monopolar device. In laparoscopic surgery, various energy devices can be used, such as monopolar devices, argon beam coagulators [8], and sealing devices. It has been reported that the use of sealing devices can make laparoscopic decortication safer and reduce the operation time [5], [9]. On the other hand, Tuncel et al. reported that performing laparoscopic decortication with a conventional monopolar device is an effective and safe treatment option for symptomatic simple renal cysts and that more expensive energy sources are not required. Although the efficacy and safety of the operation are utmost importance, it is also very important to perform the operation at low cost, considering the financial toxicity of the patient or the hospital. In our case, we showed that the laparoscopic decortication of renal cyst using conventional monopolar device, which is inexpensive, can be used to perform the effective and safe treatment.

## 4 Conclusion

Our case has shown that laparoscopic decortication without using expensive energy sources is effective and safe treatment for symptomatic large simple renal cyst.

## Sources of funding

None.

## Consent

Written informed consent was obtained from the patient for publication of this case report and accompanying images. A copy of the written consent is available for review by the Editor-in-Chief of this journal on request.

## Guarantor

Kota Kobayashi, MD.

## Provenance and peer review

Not commissioned, externally peer-reviewed.

## Ethical statement

Institutional Board Review approved this study.

## Research registration

None.

## CRediT authorship contribution statement

Kota Kobayashi performed operation, wrote the manuscript.

Eren Iwasa, Daisuke Yamashita and Akitoshi Takizawa performed operation.

Other authors wrote and checked the manuscript.

## Declaration of competing interest

The authors declare that no conflicts of interest exist.
